# Targeted Killing of *Pseudomonas aeruginosa* by Pyocin G Occurs *via* the Hemin Transporter Hur

**DOI:** 10.1016/j.jmb.2020.04.020

**Published:** 2020-06-12

**Authors:** Iva Atanaskovic, Khedidja Mosbahi, Connor Sharp, Nicholas G. Housden, Renata Kaminska, Daniel Walker, Colin Kleanthous

**Affiliations:** 1 Department of Biochemistry, University of Oxford, South Parks Road, Oxford OX1 3QU, UK; 2 Department of Zoology, University of Oxford, 11a Mansfield Rd, Oxford OX1 3SZ, UK; 3 Institute of Infection, Immunity, and Inflammation, College of Medical, Veterinary, and Life Sciences, University of Glasgow, G12 8QQ Glasgow, UK

**Keywords:** bacteriocin, protein antibiotic, protein import, TonB-dependent transporter, FtsH, PMF, proton motive force, PyoG, pyocin G, TBDT, TonB-dependent transporter, β-OG, *n-octyl-β-d-glucoside*, PBS, phosphate-buffered saline

## Abstract

*Pseudomonas aeruginosa* is a priority pathogen for the development of new antibiotics, particularly because multi-drug-resistant strains of this bacterium cause serious nosocomial infections and are the leading cause of death in cystic fibrosis patients. Pyocins, bacteriocins of *P. aeruginosa*, are potent and diverse protein antibiotics that are deployed during bacterial competition. Pyocins are produced by more than 90% of *P. aeruginosa* strains and may have utility as last resort antibiotics against this bacterium. In this study, we explore the antimicrobial activity of a newly discovered pyocin called pyocin G (PyoG). We demonstrate that PyoG has broad killing activity against a collection of clinical *P. aeruginosa* isolates and is active in a *Galleria mellonella* infection model. We go on to identify cell envelope proteins that are necessary for the import of PyoG and its killing activity. PyoG recognizes bacterial cells by binding to Hur, an outer-membrane TonB-dependent transporter. Both pyocin and Hur interact with TonB1, which in complex with ExbB–ExbD links the proton motive force generated across the inner membrane with energy-dependent pyocin translocation across the outer membrane. Inner-membrane translocation of PyoG is dependent on the conserved inner-membrane AAA + ATPase/protease, FtsH. We also report a functional exploration of the PyoG receptor. We demonstrate that Hur can bind to hemin *in vitro* and that this interaction is blocked by PyoG, confirming the role of Hur in hemin acquisition.

## Highlights

• Characterization of pyocin G, a new protein antibiotic of *Pseudomonas aeruginosa*
• Exploration of cell envelope proteins that import pyocin G
• Functional characterization of Hur, the pyocin G receptor
• Demonstration of competitive binding of hemin and pyocin G to Hur

## Graphical abstract

**Figure:**
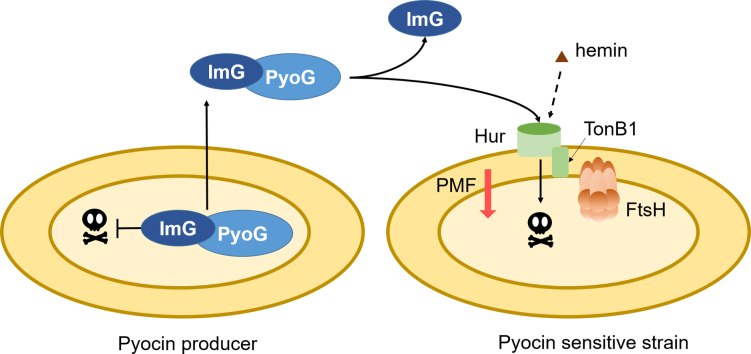

## Introduction

Antibiotic-resistant *Pseudomonas aeruginosa* is a common cause of lung and wound infections in immunocompromised patients. The World Health Organisation lists this organism as a pathogen of critical priority for the development of new antibiotics [1]. Pyocins are protein antibiotics with narrow-spectrum activity that could be developed into new antimicrobials for treating *P. aeruginosa* infections, as demonstrated in various *in vivo* models [2]. Pyocins belong to a larger group of proteinaceous molecules called bacteriocins, which are used by bacteria to compete for resources by killing competitors, usually of the same bacterial species. Pyocins are multi-domain proteins produced in combination with an immunity protein. The immunity protein binds to the cytotoxic domain at the pyocin C terminus to inhibit its activity and protect the producing strain from the lethal effects of the pyocin. Once released by producing strains, the N-terminal sequences of pyocins interact with proteins in the cell envelope in order to assemble energized import machineries called translocons [3]. Multiple protein–protein interactions are required for pyocin import, which explains their narrow-spectrum killing activity and so defining how these membrane-associated translocons are assembled aids in their development as species-specific antimicrobials [2].

Different pyocins target different cell envelope components in order to kill *P. aeruginosa*. Nuclease pyocins translocate to the cytoplasm to degrade nucleic acids. To do so, they first bind an outer-membrane receptor, which can be common polysaccharide antigen [4] or an outer-membrane protein [5,6]. They next have to exploit an outer-membrane protein translocator, usually by hijacking a nutrient importer. The energy source for translocation is the proton motive force (PMF) across the inner membrane. In general, the PMF and translocation are linked through the TonB–ExbD–ExbB system in *P. aeruginosa* [7]. Finally, nuclease pyocins likely also require an inner-membrane translocator to enter the cell, but no such import route has been identified in this organism.

Pyocins show high strain specificity by targeting particular receptors and translocators in the outer membrane [8]. Some pyocins share homology at the N terminus, which implies they use the same receptor to deliver different cytotoxic domains into the cell [9]. Pyocins sharing the same receptor are treated as members of the same pyocin group. Strain coverage of a pyocin depends on how widespread its receptor is in *P. aeruginosa* genomes. Therefore, an important requirement for the potential use of pyocins in the clinic is to pair up different pyocin groups with their receptors. If these pairs are known, it can also be predicted from genomic data which strains of *P. aeruginosa* are likely to be sensitive to which pyocins.

A limitation in the future biomedical use of pyocins is the occurrence of pyocin resistance mechanisms. *P. aeruginosa* is intrinsically resistant to all pyocins it produces, since it will also have immunity protein genes for them. Additionally, resistance can occur through spontaneous mutations affecting proteins required for pyocin translocation. These problems can, however, be overcome through the use of cocktails comprising toxins that have different cell envelope targets and different immunity proteins. Mining *P. aeruginosa* genomes for novel pyocin genes is therefore an important step in developing this class of antibiotics as therapeutics. We have developed a bioinformatics pipeline for searching genomes of different bacteria for nuclease bacteriocins, which represents a valuable tool for exploring the diversity of pyocins [9]. The pipeline led to the discovery of several new putative pyocin genes, including pyocin G (PyoG). PyoG has an N-terminal region homologous to previously described pyocins from the S1 group (S1, S13 or SD1 and S6) and a cytotoxic domain homologous to carocin D, an endonuclease bacteriocin from *Pectobacterium carotovorum* [10]. In this study, we expressed and purified this newly identified pyocin, and demonstrated its cytotoxic activity against clinical isolates of *P. aeruginosa* and its efficacy in a *Galleria mallonella* infection model. We also determined the cell envelope components necessary for translocation of the pyocin, showing that pyocins from the S1 group share a common receptor, Hur (*h*emin *u*ptake *r*eceptor). We further show that Hur has a role in hemin acquisition and hence may have a role in *P. aeruginosa* virulence.

## Results and Discussion

### PyoG is a novel pyocin from the S1 group

**Figure S1:**
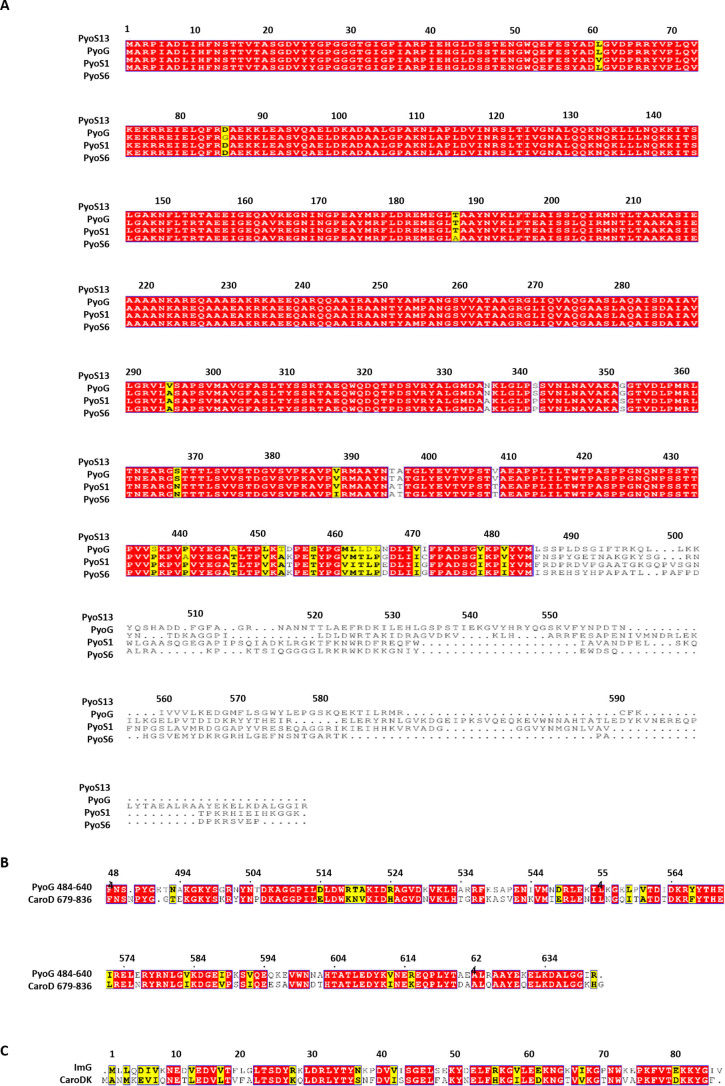
Pyocin G has an N-terminal region conserved among other S1-group pyocins, and a distinct cytotoxic domain. (A) Alignment of representative sequences of S1-group pyocins. (B) alignment of the cytotoxic domain of pyocin G and carocin D. (C) Alignment of the pyocin G (ImG) and carocin D (caroDI) immunity protein.

**Figure S2:**
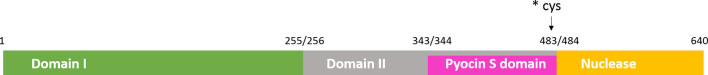
Functional domain organization of pyocin G. Domains were annotated based on sequence homology to pyocin S1 and carocin D. Pyocin S domain (PFAM domain PF06958) was annotated by SMART. Position of cysteine introduced for fluorescence labeling is indicated with an arrow.

PyoG is a 640-amino-acid toxin that belongs to the S1 group in which the first 483 residues are similar to pyocins S1, S6 and S13 (Figure S1). This suggests that all S1-group pyocins share the same cell entry route since pyocin translocation domains are generally located at the N terminus [5,6,11]. However, this entry mechanism is currently unknown. We first functionally annotated the domains of PyoG by sequence homology with other bacteriocins. Sano *et al*. have previously determined two functional domains in the conserved N-terminal region of pyocin S1 (Figure S2, domains I and II) and linked them to receptor binding and cell import albeit that the proteins involved were not identified [12]. Residues 1–483 are predicted to be helical, which is the case for the translocation domains of other pyocins for which structures have been reported [5,6]. The cytotoxic domain, and its corresponding immunity protein, is not homologous to any other pyocin but to carocin D (Figure S1). Carocin D is a nuclease bacteriocin from *P. carotovorum* that shows DNase activity *in vitro* [10], implying PyoG is also a nuclease. This is supported by the presence of the Pyocin S domain (Figure S2), a conserved domain shared among all nuclease bacteriocins, yet missing from pyocins, which attack the cell envelope [9]. The function of this domain has yet to be determined, but is thought to be linked to the inner-membrane translocation step in gram-negative bacteria.

**Figure 1:**
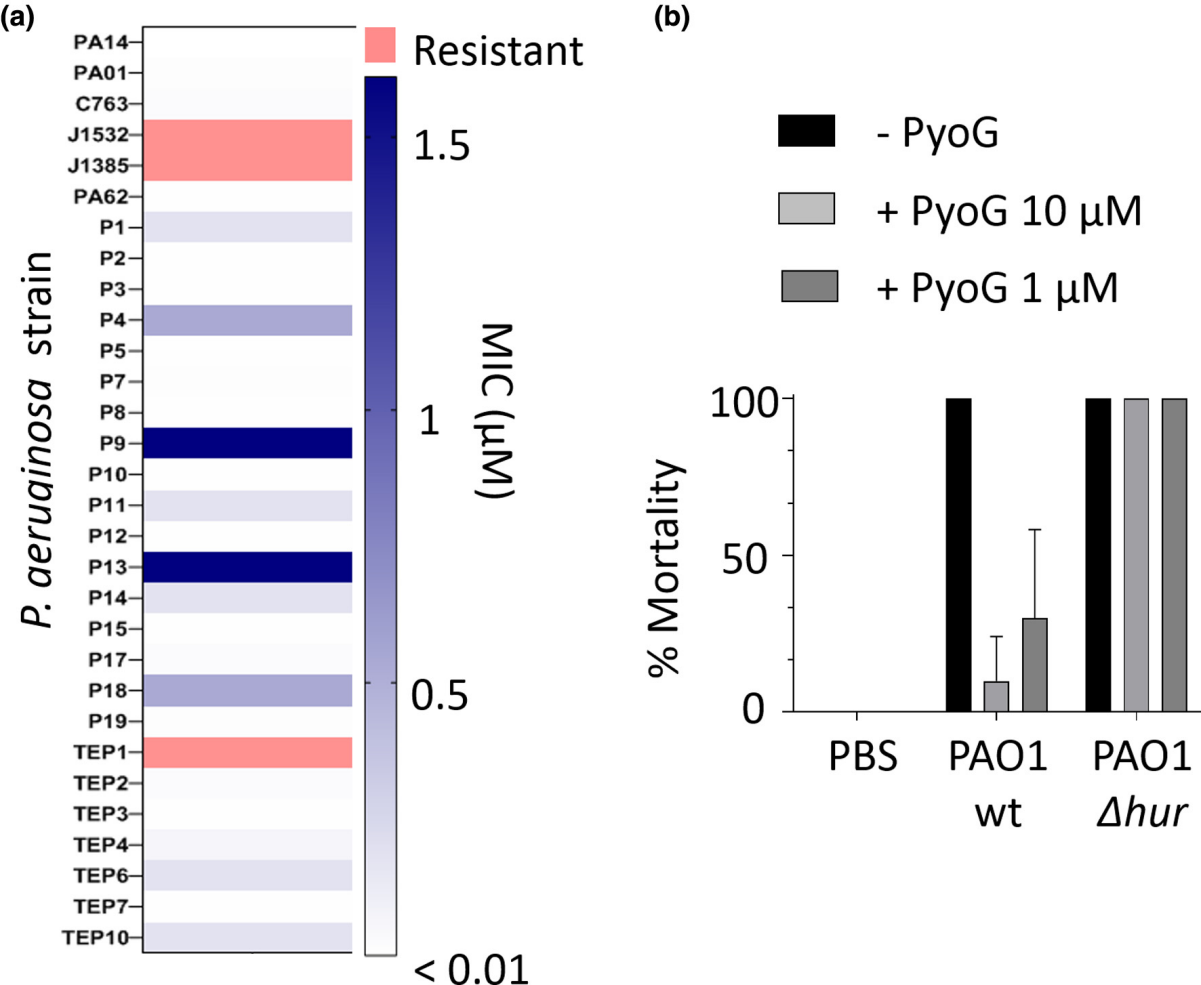
PyoG is active against clinical isolates of *P. aeruginosa* and can rescue *G. mallonella* from a lethal dose of *P. aeruginosa* PAO1. (a) PyoG MICs for a collection of *P. aeruginosa* clinical isolates. Resistant strains are shown in red, and MICs values are represented as shades of blue. Most strains are sensitive to the pyocin. (b) *In vivo* activity of PyoG against a lethal dose of *P. aeruginosa* PAO1 in the *G. mallonella* infection model. PAO1 *Δhur* is a PyoG-resistant mutant which lacks the receptor for the pyocin.

**Figure S3:**
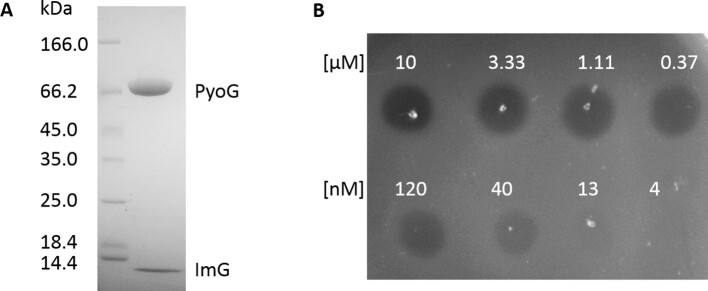
Purification and activity of PyoG. (A) SDS/PAGE gel (4%–16 %) of purified PyoG (PyoG, 69 kDa) and its immunity protein (ImG, 10 kDa). (B) Spot killing assay against *P. aeruginosa* PAO1 shows that the purified pyocin is active and has an MIC in the nM range.

We heterologously expressed PyoG in complex with its immunity protein ImG and assessed its activity (Figure S3). Purified PyoG killed *P. aeruginosa* PAO1 with a nanomolar MIC (Figure S3B). To assess *P. aeruginosa* strain coverage, we tested the activity of PyoG against a collection of 32 *P. aeruginosa* clinical isolates. One of the factors that influences bacteriocin strain coverage is immunity. Strains that produce a pyocin have the corresponding immunity gene in the same operon, but immunity protein genes can also be orphans. If the PyoG immunity is not being produced by many *P. aeruginosa* strains, then it would be anticipated to have a good strain coverage. PyoG immunity is present in 11 of 6973 strains in the PubMLST Database [13]. This is a small number of strains when compared to the pyocin S1 immunity, which is present in 744 strains. Indeed, 90% of screened strains were sensitive to PyoG, where most had nanomolar MICs (Figure 1(a)). Good strain coverage also indicates that the PyoG translocation machinery is common in *P. aeruginosa* genomes.

We also tested the efficacy of PyoG against *P. aeruginosa* strains using a *G. mallonella* infection model. *G. mallonella* was exposed to a lethal dose of *P. aeruginosa* PAO1. Three-hour post-infection larvae were treated with PyoG, which led to rescue of larvae (Figure 1(b)). In conclusion, PyoG is a newly discovered pyocin from the S1 group, which shows good strain coverage and *in vivo* activity against *P. aeruginosa* infection.

### Defining the translocation machinery for PyoG

**Figure 2:**
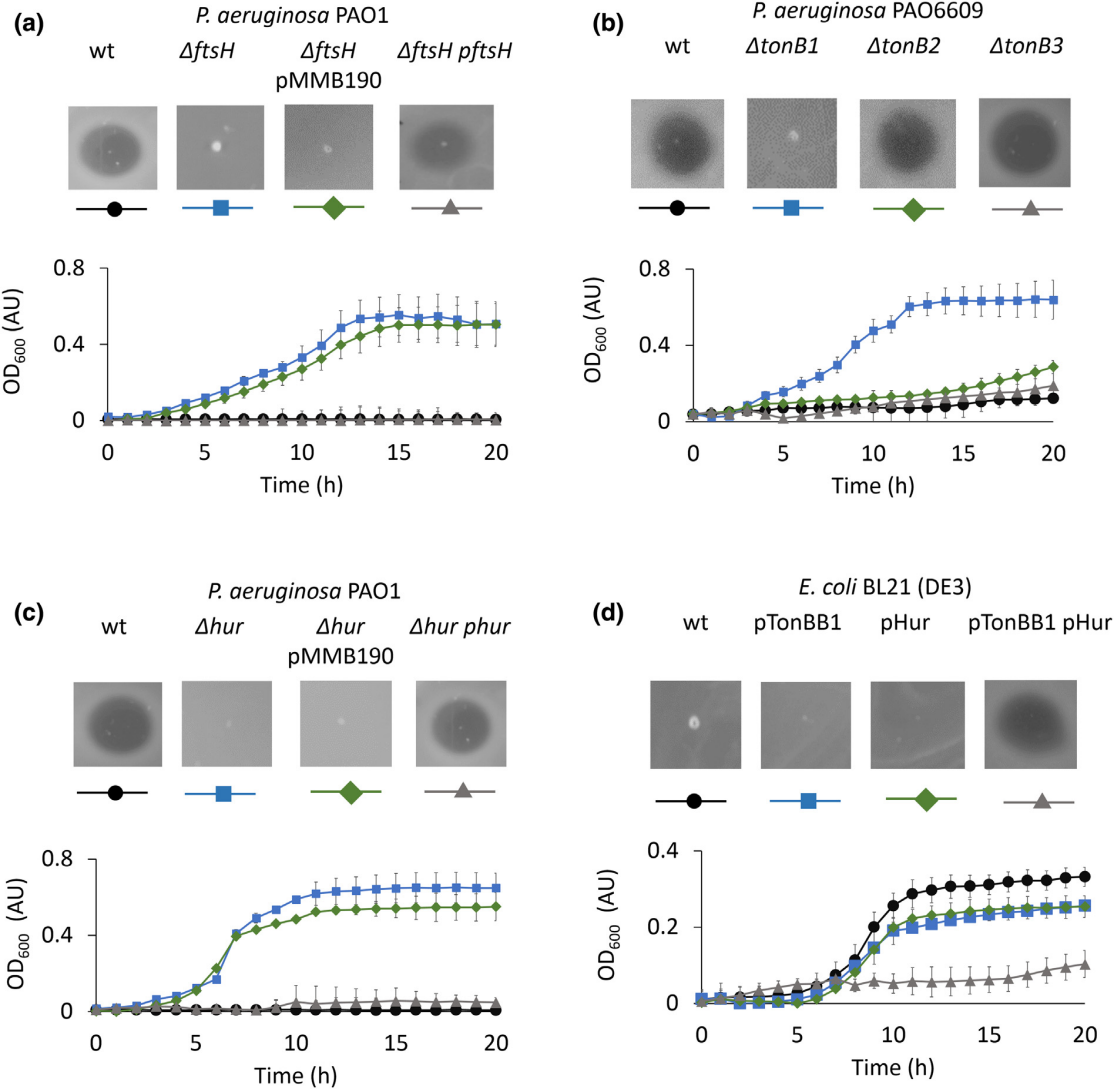
Plate and liquid killing assays with 10 μM PyoG reveal which cell envelope proteins are involved in its import. For OD_600_, mean of three biological replicates with standard deviations is shown. (a) FtsH, an inner-membrane AAA + ATPase/protease, is necessary for PyoG killing activity. *P. aeruginosa* PAO1 *ΔftsH* is resistant to PyoG and is unaffected by the introduction of the empty shuttle vector pMMB190 (*ΔftsH* pMMB190). However, transformation of PAO1 ΔftsH with *pftsH* complemented the *ftsH* deletion and restored sensitivity to PyoG. (b) TonB1, a protein that links the PMF generated on the inner membrane with translocation across the outer membrane, is necessary for PyoG killing activity. *ΔtonB1* mutant of *P. aeruginosa* is resistant to PyoG, while *ΔtonB2* and *ΔtonB3* mutants are sensitive. (c) The killing activity of PyoG depends on an outer-membrane transporter, Hur. Deletion of *hur* induces resistance to the pyocin. Sensitivity can be restored if *hur* is complemented from a plasmid. *Phur* is *hur* cloned from PAO1 into pMMB190. (d) *E. coli* BL21 (DE3) is not sensitive to PyoG. PyoG sensitivity in this organism can be induced if transformed with both pTonBB1 and pHur. pTonBB1 is *E. coli* TonB^1–102^ translationally fused to *P. aeruginosa* TonB1^201–342^ and cloned into pACYCDuet-1 [6]. pHur is *hur* with the *E. coli* OmpF signal sequence, codon optimized for expression in *E. coli* and cloned into pET21d.

We first investigated if any of the cell envelope proteins employed by other nuclease bacteriocins are also necessary for PyoG import. One such conserved protein is the inner-membrane AAA + ATPase/protease FtsH. FtsH is required for inner-membrane translocation and proteolytic processing of the nuclease domains of several colicins that kill *Escherichia coli* cells [14]. It is not known if FtsH is important for nuclease pyocin toxicity in *P. aeruginosa*. We therefore tested PyoG killing activity against an *ftsH* deletion mutant of *P. aeruginosa* PAO1. The mutant was resistant to PyoG and sensitivity could be restored by complementation from plasmid-expressed *ftsH* (Figure 2(a)). Previous studies have shown that the protease and ATPase activities of FtsH are both required for colicin activity [14] and that proteolytic processing of colicin D and E3 nuclease domains during import depends on FtsH [15]. It is therefore likely that nuclease pyocins similarly require an FtsH-dependent cleavage step during inner-membrane transport, but this remains to be experimentally demonstrated.

**Figure 3:**
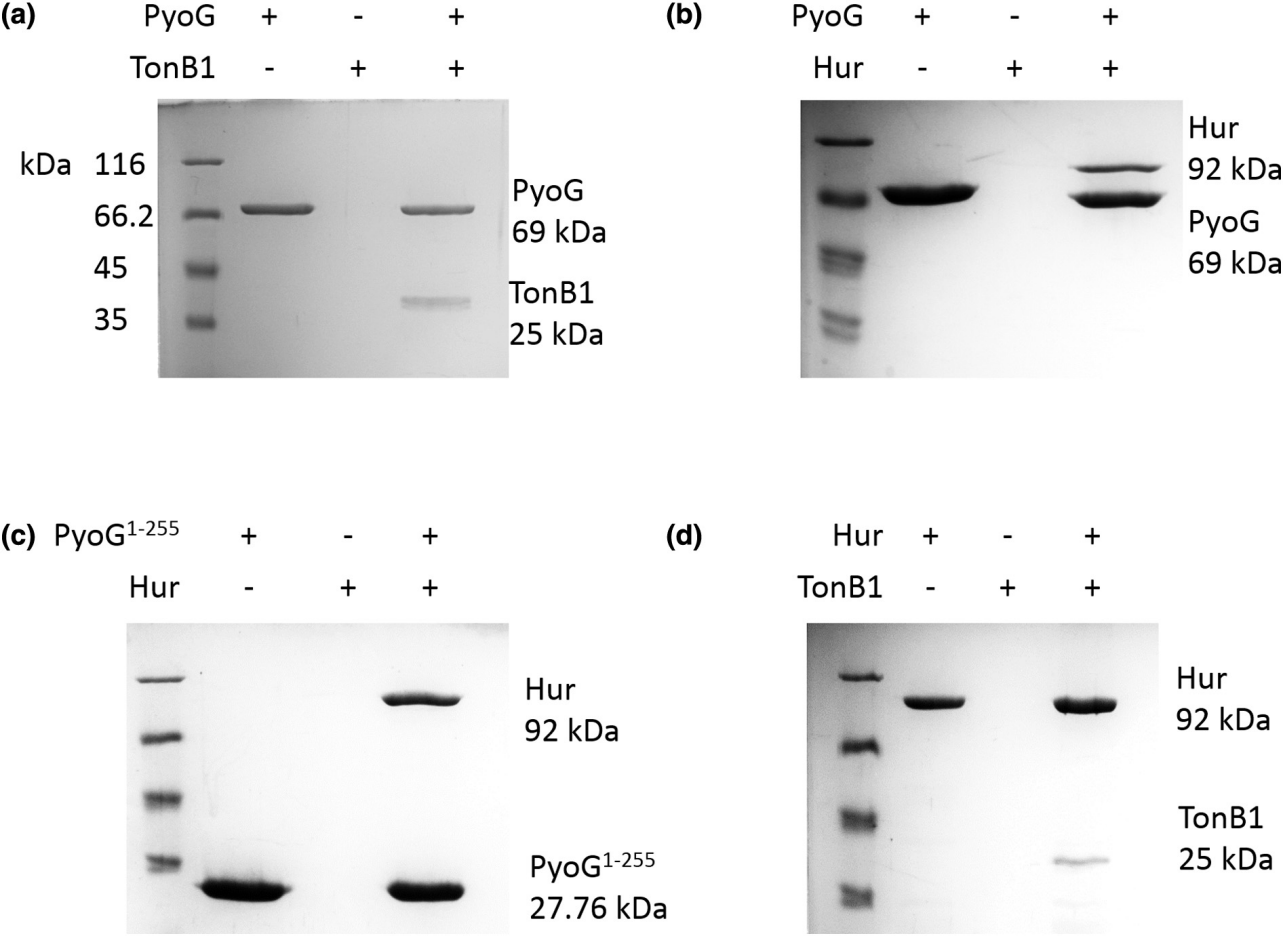
Pull-downs of PyoG translocon components. Proteins were mixed at equimolar concentrations and bound to nickel beads, which were then washed of unbound protein. Eluate content was analyzed on 12% SDS-PAGE gels. A protein marker is shown in the first lane on each gel. Eluate of the bait protein is in the second lane. Eluate of the prey protein is shown in the third lane and it verifies that the prey does not bind to beads on its own. Eluate for the mix of bait and prey is shown in the fourth lane. Positions of proteins with their molecular masses are labeled on the right side of the gel. (a) PyoG and the periplasmic region of TonB1 interact *in vitro*. (b) PyoG binds to Hur, confirming its involvement in the import of the pyocin. (c) The first 255 residues of PyoG, which are conserved among S1-group pyocins, bind to Hur. (d) Hur binds to the periplasmic region of TonB1, confirming that this is a TonB1-dependent transporter.

An inner-membrane protein system that is also known to be involved in pyocin import is the Ton system [11]. It is composed of TonB, ExbB, and ExbD and it transduces the PMF to nutrient transporters in the outer membrane. *P. aeruginosa* PAO1 has three TonB proteins: TonB1 (PA5531), TonB2 (PA0197) and TonB3 (PA0406). TonB1 and TonB2 have roles in iron acquisition and signaling [7], while TonB3 is involved in twitching motility and assembly of extracellular pili [16]. We screened transposon mutants for all three genes and found that the TonB1 mutant was resistant to PyoG (Figure 2(b)). To confirm TonB1's role in PyoG import, we purified the periplasmic domain of TonB1 to test its binding to PyoG. We could demonstrate that TonB1 and PyoG interact *in vitro via* a pull-down assay (Figure 3(a)). Therefore, PyoG depends on TonB1 for its energized import into cells.

The identification of TonB1 as a requirement for PyoG import provided the starting point for finding its outer-membrane receptor/translocator. The extended periplasmic region of TonB1 binds to outer-membrane receptors, which are usually linked to iron acquisition [7]. Such receptors are called TonB-dependent transporters (TBDTs) [17]. These are all β-barrels that share a conserved plug domain (Pfam domain PF07715) that occludes the β-barrel and a TonB binding box. Since other pyocins hijack TBDTs [5], we assumed that this was also the case of PyoG. By searching the *P. aeruginosa* PAO1 genome for TonB plug domains, we found 35 candidate receptors/translocators for PyoG (Supplementary Table 1). We next screened a library of transposon mutants for all 35 receptors and found that a transposon insertion into locus PA1302 yielded resistance to PyoG (Figure 2(c)). Sensitivity to PyoG was restored if the mutation was complemented from a plasmid, suggesting this locus codes for the PyoG receptor and/or translocator.

PA1302 has previously been linked with the import of pyocin PaeM4 in *P. aeruginosa* [18] and with hemin uptake [19]. Therefore, we named the receptor Hur (*H*emin *u*ptake *r*eceptor), which we elaborate below. To test if TonB1 and Hur are functionally linked to PyoG import, we transformed these components of the translocon into *E. coli* using the strategy developed by Behrens *et al*. [6]. We found that *E. coli* was sensitive to PyoG when co-transformed with Hur and a chimera of *E. coli* TonB and periplasmic *P. aeruginosa* TonB1 (Figure 2(d)). This indicates that Hur and periplasmic TonB1 are *P. aeruginosa*-specific components of the outer-membrane translocation machinery, with FtsH being involved in inner-membrane translocation.

**Figure 4:**
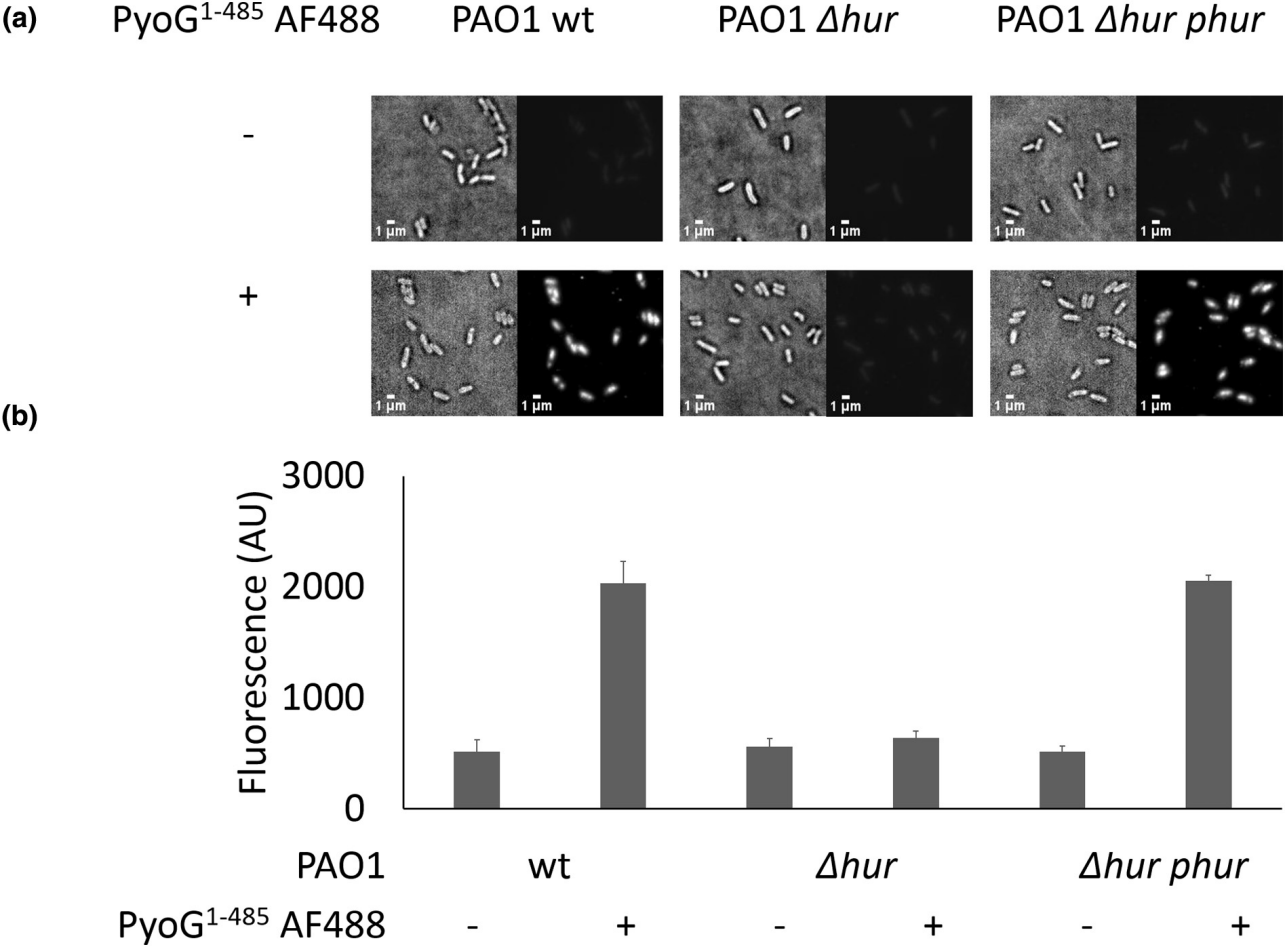
Hur is required for PyoG to target *P. aeruginosa* cells. (a) Fluorescent labeling of PAO1 wt, *Δhur* and *Δhur* complemented with Hur expressed from a plasmid (*Δhur phur)*. PyoG^1–483^, conjugated to AF488 *via* a C-terminal cysteine, was used for labeling. This construct labels the wt strain, but there is loss of labeling if *hur* is deleted. Labeling is restored if *hur* is complemented from a plasmid. Representative micrographs for each strain are shown. (b) Average fluorescence intensities for 100 cells in the presence and absence of fluorescent PyoG. Mean of three biological replicates with standard deviations is shown.

**Figure S4:**
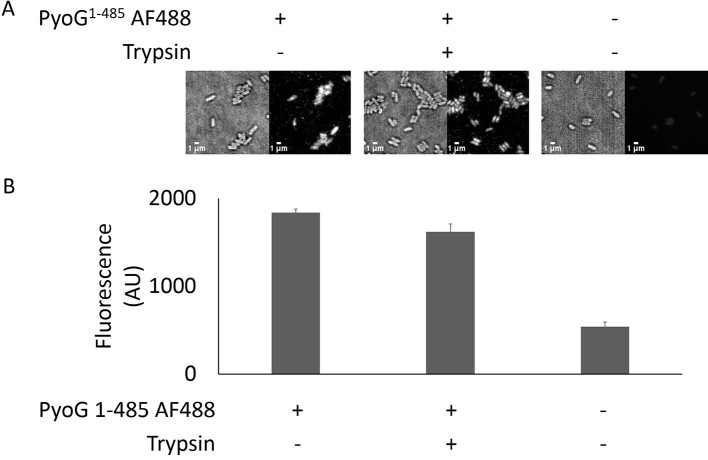
Trypsin protection assay shows that PyoG^1-485^ AF488 gets translocated across the outer membrane. (A) Representative micrographs of cells treated with trypsin after labeling with fluorescent PyoG. Cells remain fluorescent after trypsin treatment, meaning that PyoG is not available for degradation on the cell surface, but protected from trypsin due to import. (B) Average fluorescence intensities of 100 cells in the presence and absence of trypsin and fluorescent PyoG. Mean of three biological replicates with standard deviations is shown.

Several pyocins have been shown previously to bind exopolysaccharides such as common polysaccharide antigen, which concentrates the toxin on the cell surface, but then require an outer-membrane protein transporter for translocation [4,6]. Such pyocins can still bind to the cell surface if the protein translocator is deleted, but this binding does not lead to pyocin import [6]. In contrast, some bacteriocins exploit one or two outer-membrane proteins for surface recognition and translocation to the periplasm [20,21]. We therefore investigated if Hur is essential for PyoG to bind to *P. aeruginosa* cells. We constructed a PyoG construct for fluorescent labeling of live *P. aeruginosa* PAO1 cells. This construct lacks the cytotoxic domain of the pyocin and has a cysteine at the C terminus for conjugation with Alexa Fluor dyes. This construct labeled PAO1 cells but could not label the *Δhur* mutant. Additionally, complementation with Hur expressed from a plasmid restored the same fluorescence levels as PAO1 (Figure 4). Moreover, the labeled PyoG is transported across the outer membrane, since it is protected from trypsin degradation (Figure S4). The data demonstrate that Hur is an essential outer-membrane component for PyoG to bind to the surface of *P. aeruginosa* cells, and be imported into the cell. Finally, PyoG sensitivity of *E. coli* expressing Hur and the TonBB1 chimera (Figure 2(d)) also implies that Hur is the only outer-membrane component to which PyoG binds and that it likely plays the role of both receptor and translocator for PyoG import.

### PyoG and hemin compete for Hur binding

Hur is a TBDT homologous to PhuR, a hemin transporter in *P. aeruginosa* [22], HasR, a hemin transporter in *Serratia marcescens* [23], and HxuC, a hemopexin transporter of *Haemophilus influenzae* [24]. Even though sequence homology of Hur and other TBDTs strongly supports its involvement in hemin acquisition, binding of Hur to this ligand has not been demonstrated. To find interaction partners for Hur and shed more light on its function, we purified the receptor to test its binding to PyoG and hemin *in vitro*. Codon optimized *hur* from *P. aeruginosa* PAO1 was cloned with the OmpF signal sequence (MKRNILAVIVPALLVAGTANA), followed by an N-terminal His_10_ tag and a TEV cleavage site for heterologous expression in *E. coli* BL21ABCF. After expression, Hur was purified from *E. coli* outer membranes (for details, see Materials and Methods). Since the His tag of Hur could be removed by the TEV protease, we could use Hur both as bait and as prey protein in pull-downs on nickel beads. This enabled us to test if PyoG and hemin both bind to Hur *in vitro*, and if PyoG could outcompete hemin for Hur binding.

We found that full length PyoG binds Hur *in vitro* (Figure 3(b)) providing further evidence of this TBDT's role. Since the N-terminal domains are conserved among S1 group pyocins and have been hypothesized to be responsible for receptor binding [12], we decided to purify the first 255 residues of PyoG and assess its ability to bind Hur. Interestingly, we found that PyoG^1–255^ binds Hur (Figure 3(c)) confirming the role of this conserved region in receptor binding. Therefore, all S1-group pyocins probably share Hur as their receptor. We could not test if the entire conserved region (1–483) of PyoG binds to Hur since PyoG^1–483^ was not soluble in detergent (in which Hur is solubilized). Finally, to confirm that Hur is a TBDT and interacts directly with TonB1, we again used the pull-down assay and we showed that Hur binds periplasmic TonB1 (Figure 3(d)).

**Figure 5:**
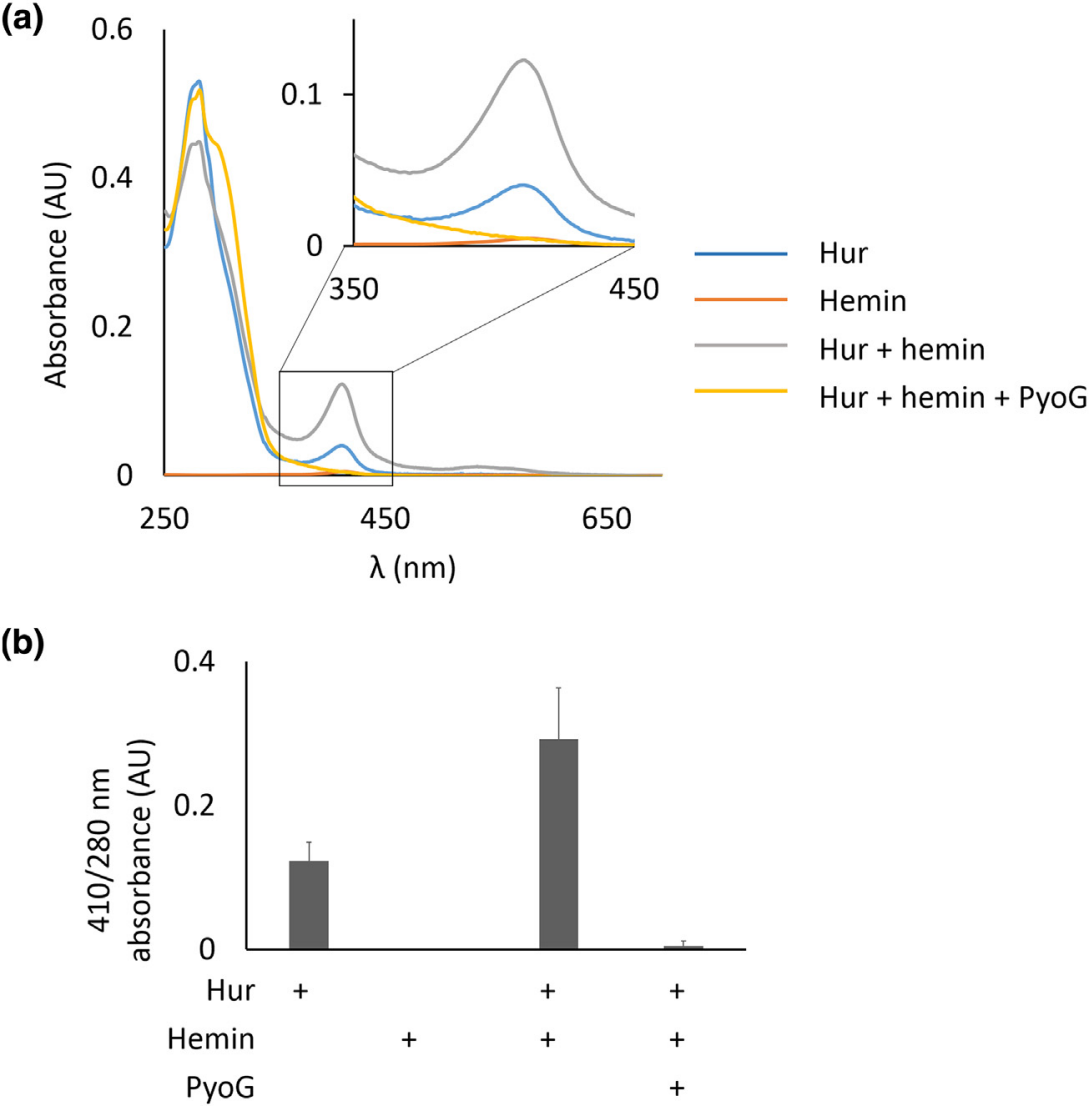
Hur binds hemin, a source of iron for *P. aeruginosa* in mammalian hosts. Hemin binding to Hur can be blocked by PyoG. (a) Pull-down of hemin with 10 μM His-tagged Hur, in the presence and absence of 10 μM PyoG lacking a purification tag. Proteins were mixed with a 100 × molar excess of hemin and bound to nickel beads. Beads were then washed of unbound protein and hemin. Absorbance spectra of eluate were measured to detect changes in the 410-nm hemin peak (enlarged in the upper corner). Representative absorbance spectra are shown. The 410-nm peak is increased if Hur is exposed to excess hemin, and no hemin peak can be observed if Hur was mixed with PyoG. No considerable 410-nm peak in the protein free control, containing hemin only, confirms that unbound hemin was washed off the beads and makes no contribution to the 410-nm absorbance in the eluate. (b) Ratio between the hemin 410-nm peak and the protein 280-nm peak in the eluate indicates that Hur binds hemin *in vitro*, which is blocked in the presence of PyoG. Mean of three technical repeats with standard deviations is shown.

**Figure S5:**
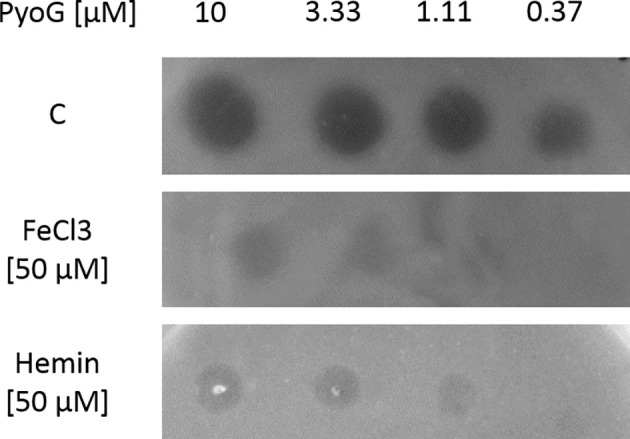
Iron and hemin inhibit the killing activity of PyoG. *P. aeruginosa* PAO1 was grown in LBA (C), in LBA supplemented with 50 μM FeCl_3_ or 50 μM hemin. A range of PyoG concentrations were spotted onto plates. The presence, clarity and size of clearance zones were inspected after overnight incubation.

**Figure S6:**
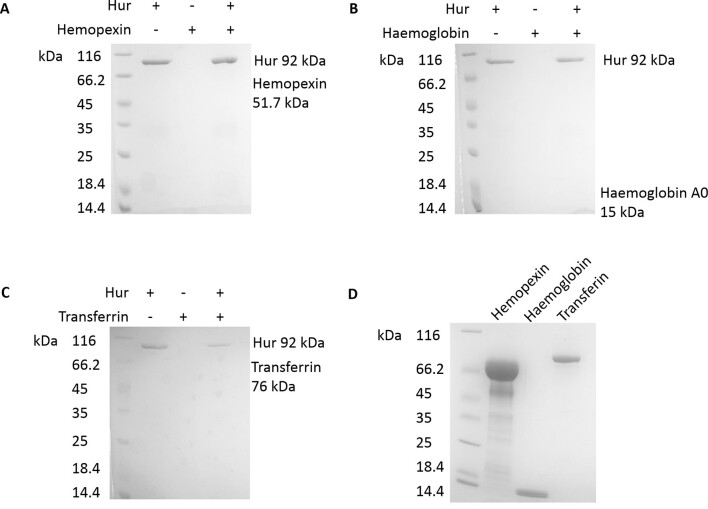
Pull-downs of His-tagged Hur and human plasma proteins. Proteins were mixed at equimolar concentrations and bound to nickel beads, which were then washed of unbound protein. The content of bead eluates was investigated on 4%–20 % SDS-PAGE gels. A protein marker is shown in the first lane on each gel. Eluate of the sample which contains just Hur is shown in the second lane, of the sample containing the plasma protein in the third, and of the sample containing both proteins in the fourth lane. Neither hemopexin (A), hemoglobin A0 (B) or transferrin (C) showed binding to Hur in this assay, since these proteins could not be detected in the pull-down eluate. (D) Stocks of human plasma proteins used as pray in the pull-down.

Otero-Asman *et al*. have shown previously that a triple PAO1 mutant lacking Hur and two other hemin uptake receptors (PhuR and HasR) has impaired growth if hemin is the sole source of iron. We found that the killing activity of PyoG is supressed in the presence of iron or hemin (Figure S5), consistent with the findings of Otero-Asman *et al*. that Hur expression is hemin dependent. These experiments indicate that Hur has a role in hemin uptake. Therefore, we deployed a spectrophotometric pull-down assay [25] to assess if purified His-tagged Hur binds hemin *in vitro*. Hur was exposed to a molar excess of hemin and then bound to nickel beads. After washing off unbound hemin, Hur was eluted and its UV–Vis spectrum recorded to assess if there was an increase at 410 nm, corresponding to the hemin peak. The ratio between the hemin and the 280-nm protein peak increased if Hur was exposed to excess hemin (Figure 5), confirming that Hur binds hemin. Because previous authors [18,19] named this receptor HxuA or HxuC (*H*emope*x*in *u*ptake receptor), we also tested *via* pull-downs if human haem containing plasma proteins could bind to the receptor. Hur could not bind to human hemopexin, hemoglobin A0 or transferrin (Figure S6). Therefore, we have renamed this protein Hur (*H*emin *u*ptake *r*eceptor), as it is more indicative of its role.

We next determined if hemin and PyoG compete for Hur binding. To test this hypothesis, we performed Hur-hemin pull-downs in the presence of PyoG lacking a purification tag. We found that PyoG depleted the amount of hemin bound to Hur in the pull-down (Figure 5). In this experiment, Hur was first exposed to hemin and then to the pyocin. PyoG can therefore displace Hur-bound hemin. These results have relevance for the clinical application of PyoG. Hur expression is upregulated under iron starvation if hemin is the sole source of iron [19]. These are the conditions that *P. aeruginosa* encounters in the host, where iron is bound to plasma proteins or as part of haem. Therefore, Hur is also upregulated in the host, as seen from expression profiles of *P. aeruginosa* isolated from human respiratory epithelia [26]. This suggests that PyoG could be active in mammalian hosts although this has yet to be demonstrated.

### Model of PyoG import mechanism into *P. aeruginosa*

**Figure 6:**
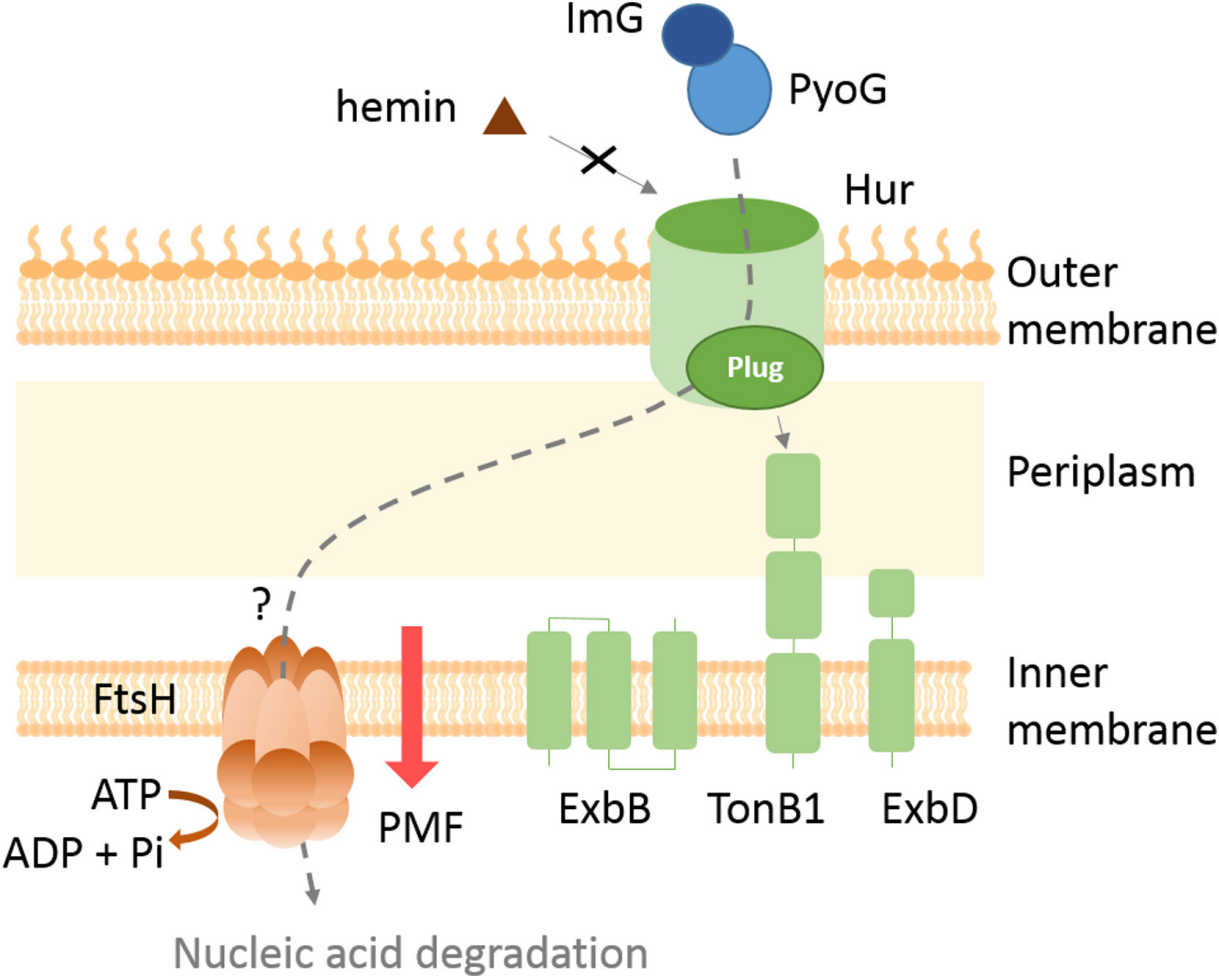
Model of PyoG translocation into *P. aeruginosa*. PyoG exploits Hur as both the receptor and the outer-membrane translocator. Binding of the pyocin to the receptor is competitive with that of hemin, its cognate ligand. Like its receptor, PyoG also binds to TonB1, which links the PMF at the inner membrane with translocation at the outer membrane. The import process, as with nuclease colicins, requires the inner-membrane AAA + ATPase/protease FtsH.

PyoG, a novel protein antibiotic of *P. aeruginosa*, exploits several cell envelope proteins in order to be imported to the cytoplasm. In this study, we identified several proteins that comprise the PyoG translocon (Figure 6). We also elaborated the link between its receptor, Hur, and hemin, the endogenous substrate.

PyoG recognizes *P. aeruginosa* by binding Hur. Hur is a TonB1-dependent transporter that has a role in hemin uptake. Binding of the pyocin and hemin to the receptor is competitive. Currently, evidence suggests that Hur is not only the receptor but also the PyoG translocator. Import of the pyocin into the periplasm depends on TonB1, which is a part of the ExbB–ExbD complex. This protein system links the proton-motive force generated across the inner membrane and the energy-dependent stages of PyoG translocation across the outer membrane. Both the receptor and the pyocin bind to TonB1 *in vitro*. Therefore, we propose that upon binding of PyoG to Hur, TonB1 dislodges the plug domain of the receptor to release the pore of the barrel, and then also pulls the pyocin into the periplasm, similar to the import mechanism of pyocin S2 through the FpvAI receptor [5]. Translocation of the pyocin through the inner membrane depends on the AAA + ATPase/protease FtsH. The pyocin could be going through the membrane channel of FtsH, which would likely requires a cleavage step during translocation, as observed for *E. coli* colicins.

## Materials and Methods

### Sequence analysis and genome searches

The bioinformatics pipeline used in the discovery of PyoG is described by Sharp *et al*. [9]. All bacteriocin sequences used in this study are deposited in the National Center for Biotechnology database under the following accession numbers: PyoG (GenBank accession no. ETV05907.1), pyocin S1 (UniProtKB/Swiss-Prot accession no. Q06583.2), pyocin S6 (GenBank accession no. KYO98147.1), pyocin S13 (National Center for Biotechnology Reference Sequence accession no. WP_023116694.1), and carocin D (GenBank accession no. ADH95192.1).

Sequence alignments were made by Clustal Omega [27] and visualized by ESPript 3.0 [28]. PFAM domains were searched for by SMART [29], and secondary structure predictions were generated by PSIPRED [30]. TonB-dependent receptors in the *P. aeruginosa* PAO1 genome were identified by HMMER 3.3 [31] using the TonB plug domain (PFAM domain PF07715).

### Strains and plasmid construction

Genes were amplified by PCR from genomic DNA or synthesized by Genewiz with codon optimization for expression in *E. coli*. All plasmids (Supplementary Table 2) were constructed by restriction enzyme digest and ligation and propagated in *E. coli* NEB5α.

All strains (Supplementary Table 1) were cultured in LB (10 g/L tryptone, 10 g/L NaCl, 5 g/L yeast extract (pH 7.2)) or M9 media (8.6 mM NaCl, 18.7 mM NH_4Cl_, 42.3 mM Na_2_HPO_4_, 22.0 mM KH_2_PO_4_, 0.4% w/v glucose, 2 mM MgSO_4_, 0.1 mM CaCl_2_) at 37 °C with shaking (140 rpm). *E. coli* BL21ΔABCF was cultured in LB Lennox (10 g/L tryptone, 5 g/L NaCl, 5 g/L yeast extract (pH 7.2)) at 30 °C with shaking (140 rpm). BL21ΔABCF was a gift from Jack Leo (Addgene plasmid #102270). *P. aeruginosa ΔftsH* was grown on LB media without addition of salt. TonB mutants of *P. aeruginosa* were grown in LB media supplemented with 100 μM FeCl_3_. All *P. aeruginosa* transposon mutants were grown in the presence of 10 μg/ml tetracycline.

Mutants of *P. aeruginosa* were complemented by transformation with a shuttle vector pMMB190 in which a gene of interest was cloned. Transformation was performed by conjugation with *E. coli* S17-1. Equal volumes of *P. aeruginosa* and S17-1 overnight cultures were mixed and incubated on top of LB agar plates for 8 h at 37 °C. Transformants of *P. aeruginosa* were then selected on 100 μg/ml carbenicilline and 10 μg/ml tetracycline.

### Protein expression, purification and quantification

All cytoplasmic His-tagged proteins (PyoG in complex with His-tagged immunity protein, PyoG^1255^, PyoG^1–483^, periplasmic domain of TonB1) were expressed in *E. coli* BL21(DE3) for 3 h in the presence of 1 mM IPTG. Cells were pelleted by centrifugation (4200***g***, 20 min), resuspended in 50 mM Tris–HCl (pH 8), 500 mM NaCl, 10 mM imidazole, 1 mM PMSF) and disrupted by sonication. Cleared lysate was loaded onto a 5-ml HisTrap FF column and eluted with a 10- to 250-mM linear gradient of imidazole. Proteins were further purified by gel filtration chromatography on a HiLoad 26/600 Superdex 200 pg column equilibrated in 50 mM Tris–HCl (pH 8), 250 mM NaCl. All columns were obtained from GE Healthcare Life Sciences.

PyoG without a purification tag was prepared by removing the His-tagged immunity protein by guanidine elution, as previously described for colicins [32]. Ten milligrams of the PyoG-ImG complex was bound to a HisTrap FF column. PyoG was then eluted in 50 mM Tris–HCl (pH 8), 250 mM NaCl, 6 M guanidine. Guanidine was removed by dialysis against 50 mM Tris–HCl (pH 8), 250 mM NaCl.

His-tagged Hur, cloned with an *E. coli* OmpF signal sequence, was expressed in BL21ΔABCF cells for 3 h in the presence of 0.1 mM IPTG. Cells were pelleted by centrifugation (4200***g***, 20 min) and disrupted by sonication in 50 mM Tris–HCl (pH 8), 1 mM PMSF. Total membranes were pelleted by ultracentrifugation at 200,000***g*** for 45 min. Inner-membrane proteins were removed in 50 mM Tris–HCl (pH 8), 2% Triton-X 100, and outer membranes were pelleted by another ultracentrifugation step. Outer-membrane proteins were extracted in 50 mM Tris–HCl (pH 8), 2% *n-octyl-β-d-glucoside* (β-OG), 5 mM EDTA. The insoluble fraction was removed by a final ultracentrifugation step, and EDTA was removed by a HiPrep 26/10 desalting column. The outer-membrane extract was then loaded onto a 5-ml HisTrap FF column equilibrated in 50 mM Tris–HCl (pH 8), 1% β-OG, 10 mM imidazole. Hur was eluted using a 10- to 500-mM linear imidazole gradient and further purified by gel filtration chromatography on a HiLoad 16/600 Superdex 200-pg column equilibrated in 50 mM Tris–HCl (pH 8), 1% β-OG. For removing the His tag, 1 mg of TEV protease was used per 10 mg of protein. TEV and uncleaved His-tagged Hur were removed using a HisTrap FF column after a 5-h incubation at room temperature.

Protein concentrations were measured by converting absorbance at 280 nm with the sequence-based predicted molar extinction coefficient (ExPASy ProtParam). Protein masses were confirmed by denaturing electrospray ionization mass spectrometry on proteins diluted in formic acid.

## Pyocin activity testing

The cytotoxic activity of PyoG was tested by a plate killing assay. *P. aeruginosa* was grown at 37 °C in LB media to an OD_600_ of 0.5. Lawns were prepared by inoculating 250 μl of culture to 5 ml of molten soft LB-agar (0.75% agar in LB), which was poured over LB-agar plates. Three-microliter drops of PyoG 3-fold serial dilutions, starting at 10 μM, were spotted onto plates. Cytotoxicity was determined by observation of clearance zones after incubation of plates at 37 °C.

Liquid killing assays were performed in 96 flat bottom plates in a 200-μl volume. LB was used as growth medium, which was supplemented with 100 μM FeCl_3_ in case of *tonB* mutants, or freed of salt in case of the *ftsH* mutant. Overnight cultures of tested strains were set to OD_600_ of 1 and diluted 100 times in fresh growth medium with or without 10 μM PyoG. OD_600_ was monitored on a CLARIOstar Plus microplate reader (BMG Labtech) on 37 °C for 20 h. All measurements were performed in triplicate.

### *Galleria mellonella* larvae infection model

*G. mellonella* larvae were obtained from Livefood UK. *P. aeruginosa* PAO1 was grown under agitation in LB broth at 37 °C to an OD_600_ of 0.6. Cells were then washed twice in sterile phosphate-buffered saline (PBS) and diluted to the desired inoculum in PBS. Inoculums were serially diluted and plated on LB agar plates just before administration for CFU counting. Groups of 10 larvae were injected with 10 μl of bacterial suspension in the hemocoel *via* the last right pro-limb or PBS as a negative control. Following challenge, larvae were placed in an incubator at 37 °C. Larvae were treated 3 h post-infection by injection of 10 μl of pyocin in the hemocoel *via* the last left pro-limb. Survival was followed for 48 h; larvae were considered dead when unresponsive to touch. Experiment was conducted in triplicate.

### Pull-down experiments

Protein pull-downs were performed either using PyoG-ImG-His6 complex or His12-Hur as a bait. Proteins were mixed to a final concentration of 10 μM in binding buffer (50 mM Tris–HCl (pH 8), 250 mM NaCl, with the addition of 1% β-OG if Hur was used). Three hundred microliters of Ni-NTA resin (QIAGEN) washed in the same buffer was added to 100 μl of protein mix. The resin was then transferred to Spin Columns (Pierce) and washed until no absorbance at 280 nm could be detected in the wash. Proteins were eluted in 100 μl of the binding buffer with the addition of 500 mM imidazole and run on a 12% SDS-PAGE gel.

Pull-downs with His-tagged Hur as bait were used to detect hemin binding. Hur (10 μM) was mixed with 1 mM hemin (Sigma) in a 250-μl reaction volume, in the presence or absence of 10 μM PyoG lacking a His tag. Ni-NTA resin (750 μl) in binding buffer was added to the mix and washed on a Spin Column until no absorbance could be detected at 280 and 410 nm, where 410 nm is the absorbance maximum for hemin in binding buffer with β-OG. Proteins were then eluted in 250 μl of binding buffer with 500 mMi midazole. Absorbance spectra of eluted proteins and hemin were measured using a V-550 UV–Visible Spectrophotometer (Jasco) in the 250- to 700-nm range. The relative amount of hemin to protein was determined by dividing the 410-nm hemin peak by the 280-nm protein peak. Experiment was performed in triplicate.

### Microscopy with fluorescently-labeled PyoG

PyoG^1–483^ with a C-terminal cysteine was covalently modified with Alexa Fluor 488 C5 Maleimide dye (Invitrogen). Pyocin of 50 μM was reduced with 10 mM DTT for 1 h at room temperature. DTT was removed on a HiTrap desalting column and protein then mixed with a 3-fold molar excess of the dye for 1 h at room temperature. The reaction was quenched with 10 mM DTT and excess dye removed using a HiLoad 16/600 Superdex 200-pg column equilibrated in 50 mM Tris–HCl (pH 8), 250 mM NaCl. Labeling efficiency, determined as described in the manufacturers protocol (Molecular Probes Inc., 2006), was typically ~ 100%.

PyoG^1–483^-AF488 was then used to label *P. aeruginosa* PAO1 wt, *Δhur* and complemented *Δhur* pHur cells. All strains were grown in M9 media until OD_600_ of 0.5. Five milliliters of cells was then washed in PBS and labeled with 2 μM PyoG^1–483^-AF488 for 30 min at room temperature. For the trypsin protection assay, after labeling cells were exposed to 0.1 mg/ml trypsin for 30 min at 37 °C as previously described [5]. All pelleting steps were performed at 7000***g*** for 3 min. Unbound dye was removed by washing in PBS, and cells were then loaded onto 1% agarose pads and imaged using an Oxford Nanoimager S microscope. Images were collected at 100-ms exposure and 20% 488-nm laser power. For every image, 100 frames were collected and averaged using the Z Project command in Image J. Average fluorescence was measured in three fields of view for a total of 100 cells per condition and was corrected by subtracting the average background fluorescence. All experiments were conducted in triplicate.

The following are the supplementary data related to this article.

Supplementary tables Image 1
