# Supplementary material for: Targeted Killing of Pseudomonas aeruginosa by Pyocin G Occurs via the Hemin Transporter Hur
Source: J Mol Biol. 2020 Jun 12;432(13):3869–80. doi: 10.1016/j.jmb.2020.04.020 (PMC7322526; doi:10.1016/j.jmb.2020.04.020)
Supplement: Supplementary file 1 — Supplementary tables [file mmc7.docx]

| Species | Strain | Characteristics | Source |
| --- | --- | --- | --- |
| *Escherichia coli* | BL21(DE3) | Expression of His-tagged bacteriocins and TonB1 | New England Biolabs |
|  | BL21_ΔABCF_ | *ΔompA*, *ΔompC*, *ΔompF*, and *ΔlamB* strain used for outer membrane protein expression | Meuskens et al. 2017.^33^ |
|  | NEB5α | Plasmid propagation | New England Biolabs |
|  | S17-1 | Conjugation with *P. aeruginosa* | American Type Culture Collection |
| *Pseudomonas aeruginosa* | PAO1 | wild type | Washington Library  Jacobs et al. 2003.^34^ |
|  | PA14 | Clinical isolate, burnwound | Lee et al. 2006.^35^ |
|  | TEP1 | Clinical isolate, sepsis | University of Glasgow, UK |
|  | TEP2 | Clinical isolate, sepsis |  |
|  | TEP3 | Clinical isolate, sepsis |  |
|  | TEP4 | Clinical isolate, sepsis |  |
|  | TEP5 | Clinical isolate, sepsis |  |
|  | TEP6 | Clinical isolate, sepsis |  |
|  | TEP7 | Clinical isolate, sepsis |  |
|  | TEP8 | Clinical isolate, sepsis |  |
|  | TEP9 | Clinical isolate, sepsis |  |
|  | TEP10 | Clinical isolate, sepsis |  |
|  | P1 | Clinical mucoid isolate, young CF patient | Royal Hospital for Sick Children, Glasgow, UK |
|  | P2 | Clinical mucoid isolate, young CF patient |  |
|  | P3 | Clinical mucoid isolate, young CF patient |  |
|  | P4 | Clinical mucoid isolate, young CF patient |  |
|  | P5 | Clinical isolate, young CF patient |  |
|  | P7 | Clinical isolate, young CF patient |  |
|  | P8 | Clinical isolate, young CF patient |  |
|  | P9 | Clinical isolate, young CF patient |  |
|  | P10 | Clinical isolate, young CF patient |  |
|  | P11 | Clinical isolate, young CF patient |  |
|  | P12 | Clinical isolate, young CF patient |  |
|  | P13 | Clinical isolate, young CF patient |  |
|  | P14 | Clinical isolate, young CF patient |  |
|  | P15 | Clinical isolate, young CF patient |  |
|  | P17 | Clinical isolate, young CF patient |  |
|  | P18 | Clinical isolate, young CF patient |  |
|  | P19 | Clinical isolate, young CF patient |  |
|  | PA7 | Clinical isolate, non-respiratory | Roy et al. 2010.^36^ |
|  | C763 | Clinical isolate, CF patient | Stewart et al. 2014.^37^ |
|  | J1385 | Clinical isolate, CF patient |  |
|  | J1532 | Clinical mucoid isolate, CF patient |  |
|  | PA62 | Environmental isolate, soil |  |
|  | PAO1 *ΔftsH* | *ftsH* deletion mutant | Lee et al. 2015.^38^ |
|  | PAO1 *ΔftsH pftsH* | PAO1 *ΔftsH* complemented with pIA30 | This study. |
|  | PAO6609 | *met-9011 amiE200 strA pvd-9* | Shirley & Lamont 2009.^7^ |
|  | PAO6609 *ΔtonB1* | *tonB1* transposon mutant |  |
|  | PAO6609 *ΔtonB2* | *tonB2* transposon mutant |  |
|  | PAO6609 *ΔtonB3* | *tonB3* transposon mutant |  |
|  | PAO1 *Δhur phur* | PA1302 transposon insertion mutant of PAO1 (PW3356) complemented with pHur | This study. |
| Transposon mutants of *P. aeruginosa* PAO1 TonB dependent transporters (TBDTs) | | Locus with transposon insertion |  |
|  | PW1255 | PA0151 | Washington Library  Jacobs et al. 2003.^34^ |
|  | PW1334 | PA0192 |  |
|  | PW1793 | PA0434 |  |
|  | PW1861 | PA0470 |  |
|  | PW2217 | PA0674 |  |
|  | PW2418 | PA0781 |  |
|  | PW2689 | PA0931 |  |
|  | PW3296 | PA1271 |  |
|  | PW3356 | PA1302 |  |
|  | PW3399 | PA1322 |  |
|  | PW3483 | PA1365 |  |
|  | PW3881 | PA1613 |  |
|  | PW4347 | PA1910 |  |
|  | PW4367 | PA1922 |  |
|  | PW9719 | PA2057 |  |
|  | PW10435 | PA2070 |  |
|  | PW4597 | PA2089 |  |
|  | PW4870 | PA2289 |  |
|  | PW4938 | PA2335 |  |
|  | PW5144 | PA2466 |  |
|  | PW5348 | PA2590 |  |
|  | PW5503 | PA2688 |  |
|  | PW5892 | PA2911 |  |
|  | PW6483 | PA3268 |  |
|  | PW6749 | PA3408 |  |
|  | PW7415 | PA3790 |  |
|  | PW7590 | PA3901 |  |
|  | PW8043 | PA4156 |  |
|  | PW7180 | PA4221 |  |
|  | PW8599 | PA4514 |  |
|  | PW8871 | PA4675 |  |
|  | PW8934 | PA4710 |  |
|  | PW9134 | PA4837 |  |
|  | PW9241 | PA4897 |  |
|  | PW10317 | PA5505 |  |

Supplementary Table 1. Bacterial strains used in this study.

| Plasmid | Characteristics | Source |
| --- | --- | --- |
| pET21d | pBR322 origin, Hist tag, Amp^r^ | NEB |
| pMMB190 | Broad-host-range cloning vector, Amp^r^, pMMB66EH, tac promoter, LacZα | Morales et al. 1991.^32^ |
| pNGH262 | His6-Im-PyoG cloned into pET21d vector at NdeI and HindIII sites | This study |
| pPW17 | His6-TEV-TonB1(109-342) from PAO1 cloned into pETM11 vector at NcoI and SacI sites | White et al. 2017.^5^ |
| *pftsH* | *ftsH* from PAO1 cloned into pMMB190 at BamHI and HindIII sites | This study |
| pTonBB1 | *E. coli* TonB1-102 translationally fused to *P. aeruginosa* TonB1201-342 cloned into pACYCDuet-1 at NdeI/XhoI sites | Behrens et al. 2020.^6^ |
| *phur* | *hur* (PA1302) from PAO1 cloned into pMMB190 at BamHI and HindIII sites | This study |
| pG1-255 | His6-PyoG1-255 cloned into pET21d vector at NdeI and HindIII sites | This study |
| pOmpF(ss)Hur | His10-TEV-Hur from PAO1 with the OmpF signal sequence cloned into pET21d vector at NdeI and HindIII sites | This study |

Supplementary Table 2. Plasmids used in this study.

**References**

[33] Meuskens, I., Michalik, M., Chauhan, N., Linke, D. &amp; Leo, J.C. (2017). A New Strain Collection for Improved Expression of Outer Membrane Proteins. Front. Cell. Infect. Microbiol. 7, 464

[34] Jacobs, M.A., Alwood, A., Thaipisuttikul, I., Spencer, D., Haugen, E., Ernst, S., Will, O., Kaul, R., Raymond, C., Levy, R., Chun-Rong, L., Guenthner, D., Bovee, D., Olson, M.V. &amp; Manoil, C. (2003). Comprehensive transposon mutant library of Pseudomonas aeruginosa. Proc. Natl. Acad. Sci. U S A 100, 14339-44.

[35] Lee, D.G., Urbach, J.M., Wu, G., Liberati, N.T., Feinbaum, R.L., Miyata, S., Diggins, L.T., He, J., Saucier, M., Deziel, E., Friedman, L., Li, L., Grills, G., Montgomery, K., Kucherlapati, R., Rahme, L.G. &amp; Ausubel, F.M. (2006). Genomic analysis reveals that Pseudomonas aeruginosa virulence is combinatorial. Genome Biol. 7, R90.

[36] Roy, P.H., Tetu, S.G., Larouche, A., Elbourne, L., Tremblay, S., Ren, Q., Dodson, R., Harkins, D., Shay, R., Watkins, K., Mahamoud, Y. &amp; Paulsen, I.T. (2010). Complete genome sequence of the multiresistant taxonomic outlier Pseudomonas aeruginosa PA7. PLoS One. 5, :e8842.

[37] Stewart, L., Ford, A., Sangal, V., Jeukens, J., Boyle, B., Kukavica-Ibrulj, I., Caim, S., Crossman, L., Hoskisson, P.A., Levesque, R. &amp; Tucker N.P. (2014). Draft genomes of 12 host-adapted and environmental isolates of Pseudomonas aeruginosa and their positions in the core genome phylogeny. Pathog. Dis. 71, 20-5.

[38] Lee, S.A., Gallagher, L.A., Thongdee, M., Staudinger, B.J., Lippman, S., Singh, P.K. &amp; Manoil, C. (2015). General and condition-specific essential functions of Pseudomonas aeruginosa. Proc. Natl. Acad. Sci. U S A. 112, 5189-94.
